# Contribution of high-technology procedures to public healthcare expenditures: the case of ischemic heart disease in Portugal, 2002–2015

**DOI:** 10.1007/s10754-024-09372-5

**Published:** 2024-03-29

**Authors:** Wenkang Ma, Ana Timóteo, Vanessa Ribeiro, Céu Mateus, Julian Perelman

**Affiliations:** 1 Department of Economics and Related Studies, University of York, York, England, UK; 2 Nova Medical School, Nova University of Lisbon, Lisbon, Portugal; 3 Comprehensive Health Research Center, Nova University of Lisbon, Lisbon, Portugal; 4 Central Administration of the Health System, Lisbon, Portugal; 5 Lancaster University, Lancaster, England, UK; 6 Nova National School of Public Health, Lisbon, Portugal

**Keywords:** Health expenditure, Medical technology, Decomposition methods, Ischemic heart disease, Portugal, I10, I18

## Abstract

The magnitude of the impact of technological innovations on healthcare expenditure is unclear. This paper estimated the impact of high-technology procedures on public healthcare expenditure for patients with ischemic heart disease (IHD) in Portugal. The Blinder–Oaxaca decomposition method was applied to Portuguese NHS administrative data for IHD discharges during two periods, 2008–2015 vs. 2002–2007 (N = 434,870). We modelled per episode healthcare expenditures on the introduction of new technologies, adjusting for GDP, patient age, and comorbidities. The per episode healthcare expenditure was significantly higher in 2008–2015 compared to 2002–2007 for IHD discharges. The increase in the use of high-technology procedures contributed to 28.6% of this growth among all IHD patients, and to 18.4%, 6.8%, 11.1%, and 29.2% for acute myocardial infarction, unstable angina, stable angina, and other IHDs, respectively. Changes in the use of stents and embolic protection and/or coronary brachytherapy devices were the largest contributors to expenditure growth. High-technology procedures were confirmed as a key driver of public healthcare expenditure growth in Portugal, contributing to more than a quarter of this growth.

**Supplementary Information:**

The online version contains supplementary material available at 10.1007/s10754-024-09372-5.

## Introduction

Medical technology advancements, such as innovative medical devices and treatment procedures, have improved the quality of medical care and thus improved health outcomes and productivity during the past decades (Cutler & McClellan, 2001; Skinner & Staiger, 2015). At the same time, technological innovations have been indicated as a crucial factor in increasing healthcare spending overall in high income countries in Europe and in the United States (US) (Chandra & Skinner, 2012; Dieleman et al., 2017; Dybczak & Przywara, 2010; Murthy & Ketenci, 2017; Neumann & Weinstein, 1991; Nghiem & Connelly, 2017; Okunade & Osmani, 2018). In a context with tight health budget constraints, the introduction of new technologies has thus been particularly scrutinized, through the development of health technology assessment (HTA) methods and their practical use, in several high-income countries, by drugs and devices regulation agencies.

However, few studies have quantified the effect of technological innovations due to lack of suitable empirical data and statistical methods, i.e., it was difficult to identify the scope of technologies to consider and evaluate their specific effect (e.g., types of technologies and what diseases they were applied to) (Chandra & Skinner, 2012; Dybczak & Przywara, 2010; Okunade & Osmani, 2018; Rodriguez Santana et al., 2020). Essentially, innovations were often considered together with other non-controllable factors (represented by proxies) and their effects could not be separated in previous modelling efforts (Abrantes-Metz, 2012; Nghiem & Connelly, 2017). A scoping review identified 11 studies published after 2010 modelling the association between technological factors and healthcare expenditure (Table S1 in Supplementary Materials summarizes the methods and outcomes for technological factors and other covariates). Using country- or region-level aggregate expenditure data for high income regions, these studies used regression analyses (with various modifications) (Abrantes-Metz, 2012; Bilgel & Tran, 2013; Murthy & Ketenci, 2017; Murthy & Okunade, 2016; Prieto & Lago-Peñas, 2012; Wu et al., 2014; You & Okunade, 2017), decomposition method (Liu, 2020), extreme bound analysis (Hartwig & Sturm, 2014), and patient demand and supplier behaviour modelling (Chandra & Skinner, 2012). Except for one study that used technology indices derived from the use of specific medical devices (You & Okunade, 2017), authors used alternate proxies for technology advancements such as time and linear trends, residuals, and R&D expenditures. Yet, all studies identified technological innovations to be a statistically significant driver for healthcare expenditure growth. Three studies estimated the effect size: Abrantes-Metz identified the contribution of technology progress to be 32.3% as the upper bound in the US (Abrantes-Metz, 2012); Nghiem and Connelly concluded that technology progress drove 4% of health expenditure increase per year among Organisation for Economic Co-operation and Development (OECD) countries, with this proportion accelerating over the study period (1975–2004) (Nghiem & Connelly, 2017); and Liu attributed 25% of the growth in diabetes treatment expenditure in Taiwan to technology innovations (Liu, 2020). These findings are however hard to compare due to variation in methods and variables (proxies) used. Measuring the precise contribution of new specific technologies to costs and outcomes is however essential, first to justify the need of regulating their adoption and diffusion, and second, as an input to the measurement of their value for money. Indeed, while the assessment of new technologies is performed before their implementation in real practice, based on clinical trials, several researchers and stakeholders have long been advocating for the use of real-world data for ex-post assessments (Garrison et al., 2007). This paper contributes to this objective, showing that administrative data can be used for this purpose. In this study, we used inpatient administrative data for patients diagnosed with IHD in Portugal discharged between year 2002 and 2015 to estimate the contribution of change in high-technology procedure use to the per episode public healthcare expenditure, using the Blinder–Oaxaca decomposition approach.

Ischemic heart disease (IHD), also named coronary artery disease (CAD) or coronary heart disease (CHD), is a leading cause for population morbidity and mortality worldwide (Institute for Health Metrics and Evaluation, 2019; Roth et al., 2020). IHD is responsible for one-third of deaths in people over 35 years of age (Nichols et al., 2014), and causes more than half of all deaths across Europe (World Health Organization/Europe 2021). Novel therapeutic procedures have significantly reduced the complications and improved patient survival and quality of life during past decades (Dababneh & Goldstein, 2022; Roth et al., 2020). New technologies were added to the standard treatment of IHD, such as coronary artery bypass grafting, coronary balloon angioplasty and thrombolysis. Other technologies that have become part of clinical practice include coronary angioplasty with bare-metal and drug-eluting stents, embolic protection devices, percutaneous ventricular support, robotic surgery, and nanotechnologies (Kandaswamy & Zuo, 2018; Lobo et al., 2017). In Portugal, the age-adjusted mortality rate of IHD has been decreasing partly due to the use of novel technologies, especially those for better patient management in the acute phase (Pereira et al., 2013). However, IHD remains the second leading cause of death in Portugal (38.40 per 100,000 by 2018) (Institute for Health Metrics and Evaluation, 2019). IHD causes large disability-adjusted life years (DALY) loss (6% of the country’s total DALYs in 2015) (Wilkins et al., 2017) and carries a significant economic burden for the Portuguese health care system (Timóteo et al., 2020).

Administrative inpatient data from the Portuguese National Health Service (NHS) hospitals include systematically collected information on patient characteristics, diagnosis, procedures, and discharge status. The healthcare reimbursement paid by the NHS to each hospital for each discharge (patient) is derived based on this information. These data thus provide an opportunity to identify any change in the use of novel therapeutic technologies and in public healthcare expenditure for treatment of IHD patients in Portugal, and any association between them. In this study, we used NHS administrative data for patients diagnosed with IHD in Portugal discharged between year 2002 and 2015 to estimate the contribution of change in high-technology procedures use to the per episode public healthcare expenditure, using the Blinder–Oaxaca decomposition approach. We used IHD for the case study considering the huge burden the disease causes in Portugal.

Our findings distinguished themselves from previous studies and added to existing knowledge in the following ways: (a) We took advantage of the administrative data that recorded patient and treatment details to derive healthcare expenditure on a per-case level, and to capture the effect of technological innovations using variables constructed directly based on use of specific high-technology procedures; (b) We identified the effect of new technologies on healthcare expenditure more precisely by focusing on a specific disease area; (c) We applied the Blinder–Oaxaca decomposition approach to quantify this effect, i.e., the contributions of new technologies to expenditure growth. To our knowledge, this is the first study that focuses on the drivers of economic burden for IHD treatment. Based on reliable data and novel analytical methods, our findings would provide information on how to measure the economic value of new medical technologies, and thus contribute to a better resource allocation in the context of technology advancements and high burden from IHD.

## Methods

### Data

We used inpatient administrative data on all discharges from all NHS hospitals, where the publicly financed health services are provided to all people living in Portugal (i.e., universal health coverage). No data is available to assess the representativeness of our sample, due to the inexistence of detailed treatment data for private hospitals. Note, however, that NHS hospitals covered two-thirds of all healthcare expenditure across Portugal for the 2002–2015 period, and that private hospitals were generally more devoted to less complex treatments, so that we expect our sample at NHS hospitals to cover most hospitalizations for cardiovascular diseases. We included all patients aged between 18 and 100 with the following principal diagnoses coded in International Classification of Diseases, 9th Revision, Clinical Modification (ICD-9-CM): acute myocardial infarction (AMI) (410.xx), unstable angina (UA) (411.1x), stable angina (SA) (413.0x, 314.1x, 413.9x), and other forms of chronic ischemic heart disease (other IHD) (414.xx, 412). These data recorded patient characteristics (age and sex), diagnoses (principal diagnosis and up to 19 secondary diagnoses), whether it was an emergency admission, treatments (up to 20 procedures), length of stay (LOS), and discharge status (whether the patient died), and administrative information (year of admission, and the name of location of the hospital).

### Healthcare expenditure

We used the per capita healthcare expenditure from the NHS perspective, employing the unit prices used for reimbursement to NHS hospitals. The Diagnosis Related Group All Patients version 21 (DRG AP21) patient classification system was used to code the inpatient and day care episodes, serving as basis for hospital financing (Administração Central do Sistema de Saúde (ACSS), 2012; Urbano & Bentes, 1990). Adapted to the Portuguese NHS from its original version for the US, DRG AP21 groups patients into homogeneous classes in terms of the clinical features (e.g., diagnosis and disease complexity) and associated resource consumption. For each DRG, official lower and upper LOS thresholds are used to determine reimbursements. That is, the amount of reimbursement for each episode is determined by the DRG code and patient LOS: (a) For short stays (below the lower LOS threshold), the day session or daily price for the specific DRG is used; (b) for stays lasting between the corresponding lower and upper LOS thresholds, the inpatient price associated with the DRG is used; (c) for stays longer than the upper LOS threshold, the inpatient price is adjusted by adding the price for additional days of hospitalisation beyond the upper threshold. Prices and LOS thresholds are publicly available through ordinances; as ordinances (and thus, prices) are regularly updated, we used for each year the ordinance that was under application (Diário da República, 2018). A natural log transformation was applied to expenditure data to account for its non-negative right-skewed nature of distribution. All prices were inflated to 2021 euros (Statista, 2022).

### New high-technology procedures

The list of high-technology procedures was determined based on published studies and expert opinion. A scooping literature review summarised the technology breakthroughs and newly approved therapeutic technologies for IHD for the period between 2002 and 2015 in Portugal (Lobo et al., 2017). The preliminary list of treatment procedures and/or medical devices derived from this study was shared with one of the authors, a practicing cardiologist, who subjectively assessed as to which were the technological breakthroughs for treatment of IHD in Portugal between 2002 and 2015. We considered the following five procedures identified using ICD-9-CM codes: Embolic protection and coronary brachytherapy (00.66), bare-metal stent (36.06), drug-eluting stent (36.07), coronary artery bypass graft surgery and percutaneous ventricular support (36.10–36.19), and thrombolysis (99.10). The use of high-technology procedures was examined in two ways: (a) If the patient received at least one of these procedures (the variable values 1 if they receive any of the five procedures, zero otherwise); (b) if the patient received any of these five procedures separately (one variable was created for each procedure, with a value one if the patient has received it, zero otherwise).

### Covariates

Other patient characteristics were included for analysis as potential drivers for healthcare expenditure growth, namely patient sex, age, and comorbidities. Using the secondary diagnoses (comorbidities) coded by physicians based on patient records’ notes, we derived the Charlson Comorbidity Index (CCI) to indicate the level of comorbidities for each record (Charlson et al., 1987). CCI has been widely accepted as a predictor of patient prognosis and mortality for longitudinal studies and with electronic health care databases (Austin et al., 2015; Bannay et al., 2016; Charlson et al., 1987)), and could also predict future healthcare expenditure (Charlson et al., 2008, 2014). Binary variables for AMI, UA, and SA were created for subgroup analysis where applicable considering the heterogeneities between these disease subtypes. Whether the admission was an emergency and whether the patient died during the admission were also considered using binary variables. The gross domestic product (GDP) value per capita of Portugal each year was included to account for the income effect (World Bank n.d.). Hospital fixed effects were included to account for the potential heterogeneities in treatment practices, efficiency, and/or physicians’ experience.

### Descriptive analysis

The following descriptive indicators were generated for all IHD patients and for each IHD subtype by year: total number of discharges across hospitals, per capita (per discharge) healthcare expenditure, percentage of patients treated by any of the high-technology procedures under analysis, and percentage of patients treated by each of the five high-technology procedures. These indicators were compared across sex, age categories, CCI score, LOS, type of admission or discharge, and type of procedure, using analysis of variance (ANOVA) analyses or chi-square tests. The time trend of average per capita healthcare expenditure per year over the study time horizon was estimated using linear regression. A significant increase in per episode public healthcare expenditure was identified during the 2007–2008 period among all patients with IHD and patients with AMI, UA, or SA, from descriptive statistics and the regression model (details on yearly change in per capital healthcare expenditure and statistical tests for yearly expenditure growth for these patients are presented in Tables S2–S5 in Supplementary Materials). We observed a few significant changes in per capita healthcare expenditure between years in these tables (at a 0.01 *p*-value threshold). Tables S2–S5 indicate a significant change in 2007 for all patients with IHD, AMI patients, and UA patients, and a significant change for SA patients in 2008 in per capital healthcare expenditure (at a 0.01 *p*-value threshold), compared to the non-significant changes in earlier years. Therefore, two time periods, namely 2002–2007 and 2008–2015 were considered adequate periods to use in decomposition analysis. Characteristics of the patients discharged in these two periods were generated and compared using t-test.

### Blinder–Oaxaca decomposition

The Blinder–Oaxaca decomposition method decomposes the mean difference in economic outcomes based on linear regression models in a counterfactual manner (Blinder, 1973; Oaxaca, 1973). It divides the outcome differential between two groups into a part that is explained by differences in group characteristics, and a residual part that cannot be accounted for by such differences in outcome determinants and thus subsumes the unobserved predictors. This technique has been applied widely in labour economics and discrimination analyses (Chen and Zhang 2018; Hassan et al., 2019; Karbeah, 2020). It has been used to understand the difference in other (continuous and unbounded) outcomes as well, such as inequalities in health (Green & Rowe, 2021; Sharaf & Rashad, 2016) and healthcare (Amporfu & Grépin, 2019). Previous studies have explained this approach (Jann, 2008; Rahimi & Hashemi Nazari, 2021). Briefly, the Blinder–Oaxaca decomposition, based on linear regressions of two groups, say A and B, intends to find how much of the mean difference in expected outcome, Y (the vector for all outcomes), is accounted for by group differences in the predictors:

$$Y_{l} = X^{\prime}_{l} \beta_{l} + \varepsilon_{l} ,E\left( {\varepsilon_{l} } \right) = 0\quad l \in \left( {A, B} \right)$$

where l is the group index, Xʹ is the transposition of X which is a vector containing the predictors and a constant, β contains the slope parameters and the intercept, and ε is the error term. The mean outcome difference can be expressed as the difference in the linear prediction at the group-specific means of the regressors,

$$R = E\left( {Y_{A} } \right) - E\left( {Y_{B} } \right) = E\left( {Y_{A} } \right)^\prime \beta_{A} - E\left( {Y_{B} } \right)^\prime \beta_{B}$$

where E(Y_A_)ʹ and E(Y_B_)ʹ are the transpositions of E(Y_A_) and E(Y_B_), respectively, and β_A_ and β_B_ contains the slopes and the intercept for group A and group B, respectively. This formula can be arranged into the form of a “twofold decomposition:”

$$R = Q + U$$

where

$$Q = \left\{ {E\left( {X_{A} } \right) - E\left( {X_{B} } \right)} \right\}^\prime \beta^{*}$$

$$U = E\left( {X_{A} } \right)^\prime \left( {\beta_{A} - \beta^{*} } \right) + E\left( {X_{B} } \right)^\prime \left( {\beta^{*} - \beta_{B} } \right)$$

attributing the outcome differences to group differences in the predictors (“quality effect”, Q) and an unexplained part which is usually attributed to discrimination and captures all potential effects of differences in unobserved variables (U). This method considers a non-discriminatory coefficient vector used to determine the contribution of the differences in the predictors (β*).

We conducted twofold Blinder–Oaxaca decomposition analyses in Stata software, version 17 (StataCorp LP, College Station, Texas). We performed a preliminary mixed effect regression analysis on natural logarithm form of per episode healthcare expenditure considering patient sex, age (alternatively, if the patient was over 65 years old), and CCI, whether the case was urgent, whether patient died during visit, and the use of one or any of the high-technology procedures. Independent variables that did not have statistical significance nor face validity were excluded from further analysis. Then we used the following frameworks (individual-level models) as the basis for Blinder–Oaxaca decomposition analyses for groups of discharges in year 2002–2007 vs. year 2008–2015.

For all IHD patients:

$$LOG\left( {EXPD} \right)_{i,h} = \beta_{0} + \beta_{1} GDP + \beta_{2} Age_{i,h} + \beta_{3} CCI_{i,h} + \beta_{4} TECH_{i,h} + \beta_{h} + \varepsilon_{i,h}$$

For AMI, UA, and SA patients (subgroup analyses):

$$LOG\left( {EXPD} \right)_{i,h} = \beta_{0} + \beta_{1} GDP + \beta_{2} Age_{i,h} + \beta_{3} CCI_{i,h} + \beta_{4} TECH_{i,h} + \beta_{h} + \varepsilon_{i,h}$$

$$LOG\left( {EXPD} \right)_{i,h} = \beta_{0} + \beta_{1} GDP + \beta_{2} Age_{i,h} + \beta_{3} CCI_{i,h} + \beta_{4} TECH1_{i,h} + \beta_{5} TECH2_{i,h} + \beta_{6} TECH3_{i,h} + \beta_{7} TECH4_{i,h} + \beta_{8} TECH5_{i,h} + \beta_{h} + \varepsilon_{i,h}$$

where i refers to individual in-patient episodes, h refers to hospitals, LOG(EXPD) is the natural logarithm form of per episode healthcare expenditure, GDP refers to the income effect, AGE is the patient’s age, CCI is patient’s comorbidity, β_h_ is the fixed effect of hospitals, TECH is a binary variable indicating if the patient was treated with at least one high-technology procedures, and TECH1–TECH5 are binary variables referring to use of each high-technology procedures considered, namely embolic protection and coronary brachytherapy, bare-metal stent, drug-eluting stent, CABG surgery and percutaneous ventricular support, and thrombolysis, respectively. Considering heterogeneities in disease symptom and treatment between AMI, UA, and SA, stratified analyses were performed. The robust option was used to correct for heteroscedasticity.

## Results

### Patient characteristics

Data for 434,870 discharges, with ~ 28,000– ~ 35,000 discharges each year between 2002 and 2015, were analysed. There were 174,203 (40.1%), 24,538 (5.6%), 38,910 (8.9%), and 197,219 (45.4%) discharges for AMI, UA, SA, and other IHD subtypes, respectively. The proportion of patients who were 65 years or older is higher among AMI or UA than among SA and other IHDs; AMI patients tended to have a higher CCI score and were more likely to die during hospitalization compared to patients with UA, SA, and other IHDs; patients with AMI or other IHDs were more likely to receive high-tech procedure treatments compared to those with UA or SA; both AMI and UA patients were more likely to be admitted through the emergency department (A&E), and have a longer LOS, compared to SA patients and those with other IHDs (Table 1).

**Table 1:** Sample characteristics by IHD subgroup

	Mean (standard error [SE]^a^)				*P* value for comparison between IHD subgroups^b^
	AMI (n = 174,203)	UA (n = 24,538)	SA (n = 38,910)	Other IHD (n = 197,219)	
% female	34.74	38.68	39.53	28.30	< 0.001
Age (years)	68.33 (0.03)	67.33 (0.08)	64.87 (0.06)	66.38 (0.02)	< 0.001
% age 65 years or older	62.86	60.85	53.81	59.01	< 0.001
CCI score	1.73 (0.00)	0.60 (0.01)	0.23 (0.00)	0.54 (0.00)	< 0.001
LOS (days)	8.06 (0.02)	5.51 (0.04)	2.07 (0.04)	3.85 (0.02)	< 0.001
% urgent admissions	92.58	85.03	29.13	27.93	< 0.001
% death during stay	10.15	1.96	0.20	1.71	< 0.001
% treated by any technology	40.76	16.55	14.26	42.25	< 0.001
% treated, by technology					
% embolic protection and coronary brachytherapy	23.70	6.81	5.73	19.92	< 0.001
% bare-metal stent	18.56	10.14	8.31	13.25	< 0.001
% drug-eluting stent	15.37	5.04	4.99	15.13	< 0.001
% CABG surgery and percutaneous ventricular support	1.65	0.42	0.10	12.48	< 0.001
% thrombolysis	5.41	0.57	0.13	0.54	< 0.001

The patient characteristics such as sex and age, patient comorbidity and length of hospital stay, and the treatments or technologies they received are presented and compared between IHD subtypes

*AMI* acute myocardial infarction; *UA* unstable angina; *SA* stable angina; *IHD* ischemic heart disease; *CCI* Charlson Comorbidity Index; *LOS* length of stay; *CABG* coronary artery bypass graft

^a^For continuous variables only

^b^*P* value was derived from ANOVA for continuous variables, e.g., age in years, and from chi-square test for categorical variables, e.g., percentage of urgent visits

There were 178,052 discharges during the 2002–2007 period, and 256,818 discharges during the 2008–2015 period. Across all IHD patients, those discharged during the latter period were older, had a higher comorbidity score, were more likely to receive at least one high-technology procedure, and were less likely to be admitted through A&E or die during hospital stays (Table 2).

**Table 2:** Sample characteristics by time period

	Mean (SE^a^)		*P* value for comparison between the periods^b^
	2002–2007 (n = 178,052)	2008–2015 (n = 256,818)	
% female	33.58	31.70	< 0.001
Age (years)	66.86 (0.03)	67.23 (0.02)	< 0.001
% age 65 years or older	60.21	60.18	0.827
CCI score	0.90 (0.00)	1.06 (0.00)	< 0.001
LOS (days)	6.49 (0.02)	4.77 (0.02)	< 0.001
% urgent visits	66.65	50.57	< 0.001
% death during stay	6.24	4.09	< 0.001
% treated by any technology	32.36	41.39	< 0.001
% treated by each technology			
% embolic protection and coronary brachytherapy	1.42	31.91	< 0.001
% bare-metal stent	20.06	11.08	< 0.001
% drug-eluting stent	2.59	21.49	< 0.001
% CABG surgery and percutaneous ventricular support	6.95	5.94	< 0.001
% thrombolysis	3.93	1.43	< 0.001

The patient characteristics such as sex and age, patient comorbidity and length of hospital stay, and the treatments or technologies they received are presented and compared between two study period

*SE* standard error; *AMI* acute myocardial infarction; *UA* unstable angina; *SA* stable angina; *IHD* ischemic heart disease; *CCI* Charlson Comorbidity Index; *LOS* length of stay; *CABG* coronary artery bypass graft

^a^For continuous variables only

^b^ *P* value was derived from ANOVA or t test for numeric variables, e.g., age in years, and from chi-square test for categorical variables, e.g., percentage of urgent visits

### Changes in healthcare expenditure and use of high-technology procedures

**Fig. 1:**
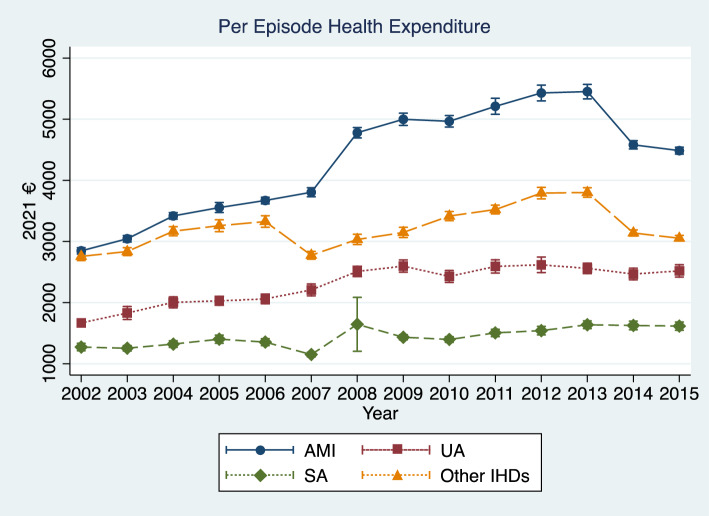
Per episode public healthcare expenditure by IHD subtype by year. *AMI* acute myocardial infarction; *UA* unstable angina; *SA* stable angina; *IHD* ischemic heart disease

AMI patients experienced the highest average expenditure, followed by other IHDs, UA, and SA (Fig. 1). Over this time, there was a clear upward trend in per episode healthcare expenditure for patients with AMI (changed from €2848 to €4486 per episode), UA (from €1668 to €2518 per episode), SA, (from €1271 to €1616 per episode), and other IHDs (from €2756 to €3052 per episode). The per episode expenditure in 2008–2015 was higher than that in 2002–2007. Note that the decline in per episode health expenditure in 2013–2014 was primarily driven by a cut in DRG prices, which resulted from the 2008–2009 economic recession and subsequent austerity measures imposed to Portugal in exchange of the financial bailout (Barros, 2012).

**Fig. 2:**
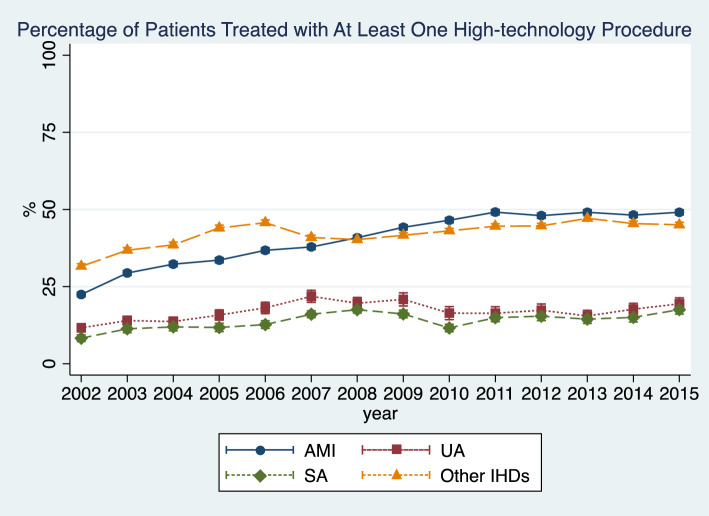
Use of high-technology procedures by IHD subtype by year. *AMI* acute myocardial infarction; *UA* unstable angina; *SA* stable angina; *IHD* ischemic heart disease

**Fig. 3:**
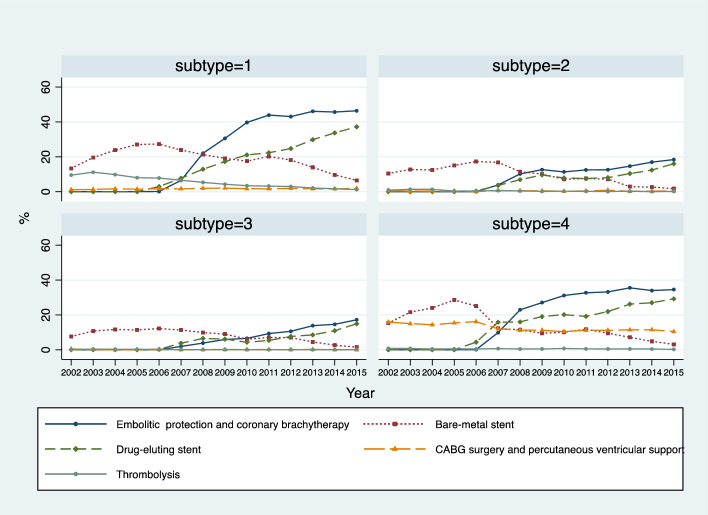
Use of each high-technology procedure by IHD subtype by year. Subtype 1–4 are AMI, UA, SA, and other IHD, respectively. *AMI* acute myocardial infarction; *UA* unstable angina; *SA* stable angina; *IHD* ischemic heart disease

Patients with AMI and other IHDs were more likely to receive high-technology procedures across all years (Fig. 2). This proportion increased over the study period for all subtypes and was the largest for patients with AMI, for whom it almost doubled (from ~ 25% to ~ 50%). Embolic protection, coronary brachytherapy, and drug-eluting stent were increasingly used over the years. Use of bare metal stents decreased. Use of CABG surgery or percutaneous ventricular support and thrombolysis remained relatively low (Fig. 3).

*Contribution of new medical technology to healthcare expenditure*

Tables 3 and 4 show the decomposition of the gap in average per episode healthcare expenditure paid to NHS hospitals between two periods, 2002–2007 (Group 1) vs. 2008–2015 (Group 2), by IHD (sub)type. Table 3 presents results of analysis where “use of any high-technology procedures” accounted for technology advancements. The average per episode healthcare expenditure was significantly higher in 2008–2015 across IHD types. Among all IHD patients, 39.6% (0.136/0.344) of the difference in healthcare expenditure (in natural logarithm form) was due to the entered variables (endowments), and 28.6% (0.098/0.344) due to use of high-technology procedures. The endowment percentages were 18.9% (0.099/0.523), 11.8% (0.047/0.400), 16.7% (0.047/0.279), and 39.1% (0.075/0.192) for patients with AMI, UA, SA, and other IHDs, respectively; the contributions of high-technology procedure were 18.4% (0.096/0.523), 6.8% (0.027/0.400), 11.1% (0.031/0.279), and 29.2% (0.056/0.192), respectively. Among all patients and patients with AMI, the use of high-technology procedures was predictive of higher healthcare expenditure.

**Table 3:** Decomposition of the difference in per episode healthcare expenditure between 2002–2007 (Group 1) vs. 2008–2015 (Group 2)

Log expenditure	All IHD		AMI		UA		SA		Other IHD	
	Prediction	*P* value	Prediction	*P* value	Prediction	*P* value	Prediction	*P* value	Prediction	*P* value
Group 1	7.527	< 0.001	7.830	< 0.001	7.223	< 0.001	6.788	< 0.001	7.445	< 0.001
Group 2	7.871	< 0.001	8.354	< 0.001	7.623	< 0.001	7.067	< 0.001	7.639	< 0.001
Differences	0.344	< 0.001	0.523	< 0.001	0.400	< 0.001	0.279	< 0.001	0.192	< 0.001
Due to endowments (explained)	0.136	0.007	0.099	< 0.001	0.047	< 0.001	0.047	0.035	0.075	0.013
GDP	0.003	0.256	− 0.002	0.174	− 0.001	0.805	0.004	0.506	0.009	0.222
Age	0.002	0.061	0.001	0.066	0.002	0.029	0.003	0.000	0.001	0.416
CCI	0.033	0.000	0.020	0.000	0.019	0	0.009	0.037	0.009	0.271
Use of any technology	0.098	< 0.001	0.096	< 0.001	0.027	0.142	0.031	0.190	0.056	0.062
Due to coefficients (unexplained)	0.208	< 0.001	0.425	< 0.001	0.353	< 0.001	0.233	< 0.001	0.117	< 0.001
GDP	0.422	0.730	− 3.707	< 0.001	− 2.899	0.032	1.715	0.512	2.032	0.244
Age	− 0.046	0.183	0.218	< 0.001	− 0.003	0.963	− 0.073	0.180	− 0.450	< 0.001
CCI	− 0.017	0.107	− 0.036	0.001	− 0.017	0.012	− 0.013	0.045	− 0.023	0.015
Use of any technology	− 0.024	0.121	− 0.087	< 0.001	− 0.035	< 0.001	0.023	0.112	− 0.009	0.653
Constant	− 0.128	0.916	3.934	< 0.001	3.308	0.016	− 1.419	0.587	− 1.394	0.424

For all IHD patients and patients diagnosed with AMI, UA, SA, and other IHDs separately, the change in per episode healthcare expenditure between the two periods are decomposed to reflect the contribution of each predictor (GDP, age, CCI, and use of technology). Negative percentage implies different direction of predictors’ effect

*AMI* acute myocardial infarction; *IHD* ischemic heart disease; *UA* unstable angina; *SA* stable angina; *CI* confidence interval; *GDP* gross domestic product; *CCI* Charlson Comorbidity Index

**Table 4:** Decomposition of the difference in per episode healthcare expenditure between two time periods, 2002–2007 (Group 1) vs. 2008–2015 (Group 2), with use of each high-technology procedure for technological factors

Log expenditure	AMI		UA		SA		Other IHD	
	Prediction	*P* value	Prediction	*P* value	Prediction	*P* value	Prediction	*P* value
Group 1	7.830	< 0.001	7.223	< 0.001	6.788	< 0.001	7.447	< 0.001
Group 2	8.354	< 0.001	7.623	< 0.001	7.067	< 0.001	7.639	< 0.001
Differences	0.523	< 0.001	0.400	< 0.001	0.279	< 0.001	0.192	< 0.001
Due to endowments (explained)	0.119	< 0.001	0.047	0.010	0.081	< 0.001	0.121	< 0.001
GDP	− 0.002	0.192	− 0.001	0.805	0.002	0.663	0.008	0.223
Age	0.000	0.075	0.002	0.031	0.003	< 0.001	0.001	0.416
CCI	0.019	< 0.001	0.019	0.000	0.009	0.037	0.008	0.271
Use of embolic protection and coronary brachytherapy	0.021	0.057	0.022	0.005	0.057	< 0.001	0.109	< 0.001
Use of bare-metal stent	− 0.046	< 0.001	− 0.070	< 0.001	− 0.034	0.029	− 0.080	< 0.001
Use of drug-eluting stent	0.121	< 0.001	0.075	< 0.001	0.047	< 0.001	0.146	< 0.001
Use of CABG surgery and percutaneous ventricular support	0.004	0.410	0.001	0.873	− 0.001	0.390	− 0.071	0.033
Use of thrombolysis	0.002	0.016	− 0.002	0.005	− 0.001	0.014	− 0.001	0.391
Due to coefficients (unexplained)	0.404	< 0.001	0.353	< 0.001	0.199	< 0.001	0.071	0.034
GDP	− 1.787	0.025	− 2.631	0.057	2.747	0.293	2.207	0.142
Age	0.182	< 0.001	− 0.004	0.953	− 0.067	0.218	− 0.420	< 0.001
CCI	− 0.037	< 0.001	− 0.018	0.013	− 0.013	0.034	− 0.021	< 0.001
Use of embolic protection and coronary brachytherapy	0.126	< 0.001	0.042	< 0.001	0.009	0.050	0.154	< 0.001
Use of bare-metal stent	− 0.138	< 0.001	− 0.052	< 0.001	− 0.026	0.003	− 0.092	< 0.001
Use of drug-eluting stent	− 0.091	< 0.001	− 0.031	< 0.001	− 0.007	0.282	− 0.117	< 0.001
Use of CABG surgery and percutaneous ventricular support	− 0.006	< 0.001	− 0.001	0.090	< 0.001	0.026	− 0.012	0.096
Use of thrombolysis	− 0.002	0.185	− 0.000	0.765	< 0.001	0.372	− 0.001	0.724
Constant	2.157	0.007	3.048	0.030	− 2.444	0.349	1.627	0.277

For all IHD patients and patients diagnosed with AMI, UA, SA, and other IHDs separately, the change in per episode healthcare expenditure between the two periods are decomposed to reflect the contribution of each predictor (GDP, age, CCI, and use of each single technology). Negative percentage implies different direction of predictors’ effect

*AMI* acute myocardial infarction; *IHD* ischemic heart disease; *UA* unstable angina; *SA* stable angina; *CI* confidence interval; *GDP* gross domestic product; *CCI* Charlson Comorbidity Index

Decomposition results for models with “use of each specific high-technology procedure” as the factor of interest were presented in Table 4. For AMI, UA, SA, and other IHD, included variables explained 22.8% (0.119/0.523), 11.7% (0.047/0.400), 28.9% (0.081/0.279), and 63.0% (0.121/0.192) of the differences in expenditure, respectively. Direction and scale of the effect of specific high-technology procedures varied across IHD subgroups. Change in the use of drug-eluting stent and bare-metal stent were the main drivers of expenditure changes, with effects in opposite directions. The use of drug-eluting stent made up 23.1% (0.121/0.523), 18.8% (0.075/0.400), 16.8% (0.047/0.279), and 76.0% (0.146/0.192) of expenditure increase for AMI, UA, SA, and other IHD patients, respectively. The use of bare-metal stents made up 8.8% (− 0.046/0.523), 17.4% (− 0.070, 0.400), 12.3% (− 0.034/0.279), and 41.7% (− 0.080/0.192) of expenditure decrease for AMI, UA, SA, and other IHD patients, respectively. Use of embolic protection and/or coronary brachytherapy was the main driver of expenditure for SA patients, explaining 20.4% (0.057/0.279) of expenditure growth.

## Discussion

### Key findings

This study identified an increase in the use of high-technology procedures among IHD patients in Portugal between 2002 and 2015. In particular, the proportion of AMI patients who were treated with at least one of the high-technology procedures doubled between this period (increased from ~ 25% to ~ 50%). In parallel, the per episode healthcare expenditure paid by the NHS also increased during the study period (by 57.6%, 51.0%, 27.1%, and 10.7% for AMI, UA, SA, and other IHDs, respectively, see Fig. 1). We identified the use of high-technology procedures as a major driver of public healthcare expenditure for IHD treatment in Portugal between 2002 and 2015, explaining 28.6% of the variation of expenditures globally, and 18.4%, 6.8%, 11.1% of that for patients with AMI, UA, and SA, respectively.

### Interpretations

NHS inpatient administrative data provided detailed information on medical procedures IHD patients received. This data allows for derivation of a proxy of the healthcare expenditure paid by the State. The trend of increase in per episode healthcare expenditure aligns with findings from recent studies across countries /regions (Achdut, 2019; Hartwig & Sturm, 2014; Murthy & Ketenci, 2017; Murthy & Okunade, 2016; Nghiem & Connelly, 2017; Wu et al., 2014) especially one study for government spending in Canada (Bilgel & Tran, 2013). We also took advantage of this data and modelled use of high-technology procedures directly, considering several technology breakthroughs for IHD treatment based on published studies and cardiology expert inputs. In this way, this study was able to generate accurate estimates for effect of technological factors compared with previous studies that used proxies for technological factors such as time trend or residual term for modelling (Meskarpour Amiri et al., 2021). Those studies widely noted the difficulty to separate the impact from technological factors from other controlled or uncontrolled factors in their analyses, and/or concluded their estimates should be treated as upper bounds of technology’s effect (Abrantes-Metz, 2012; Nghiem & Connelly, 2017). A recent study using residual as the proxy concluded that contribution of technology innovations to healthcare expenditure growth in the US was ~ 32% at most, lower than another estimate at ~ 50% using a similar dataset (Abrantes-Metz, 2012).

Our study also found that the use of any high-technology procedure contributed most to per capita expenditure growth for patients with AMI (18.4% for AMI compared to 6.8% for UA and 11.1% for SA). Considering AMI is a life-threatening event, with a high post-event mortality rate and a close to 50% hospitalisation rate within the same year (Mechanic et al., 2022), it is expected that the technology use plays a more important role for AMI patients. This also aligns with our findings in descriptive analysis that AMI patients have higher per episode healthcare expenditure in all years than patients with other IHD subtypes.

The introduction of stents was a major driver of healthcare expenditure for AMI cases, with contradictory effect for drug-eluting stents (positive effect, 23.1% of expenditure change) and bare-metal stents (negative effect, − 8.8% of expenditure change). Descriptive findings showed an increase in the use of drug-eluting stents and a decrease in that of bare-metal stents. Together, these suggest a replacement effect between different stent procedures for treatment of IHD patients in Portugal during the study period. This trend is plausible considering that the use of drug-eluting stents has been shown to be more effective in the prevention of restenosis and repeat revascularization compared to bare-metal stents, and that newer-generation drug-eluting stents have been developed to further improve efficacy and safety (especially to reduce the rate of stent thrombosis) (Bønaa et al., 2016; Cohen, 2019). Similar trends were observed among patients with UA (drug-eluting stents contributed to 18.8% of expenditure change and bare-metal stents −17.4%) which is often taken as a warning sign of an infarction episode and requires immediate treatment in hospital which could involve medicines and surgical procedures (Mayo Clinic, 2021).

In addition, the use of embolic protection and/or coronary brachytherapy was the most important single driver for the expenditure increase among SA patients, explaining 20.4% of the increase between the two periods. This aligns with the treatment practice for SA, which includes a beta-blocker or a rate-limiting calcium blocker medicine, considering its comparatively less severe nature compared to AMI and UA. Intrusive technologies are less used for SA patients (National Institute for Health & Care Excellence, 2022).

### Limitations and strengths

This study is not without limitations. First, administrative data does not include detailed clinical information, creating possible sources of bias. For example, multiple risk scores are approved in Portugal for prediction of worse prognosis for patients with AMI-related complications (Gil et al., 2019). High risk scores in AMI patients might have encouraged the prescription of advanced technologies by clinicians and led to patient’s longer LOS, but these risk scores were not included in administrative data. Second our data allowed to measure a proxy of the costs from the NHS perspective, but the real costs may have been higher if there have been regular financial bailouts resulting from public hospitals’ being underbudgeted. Such bailouts have been repeatedly observed in Portugal, and they are not considered in the official tariffs. This limitation highlights the unfortunate absence of an adequate accounting system at NHS hospitals.

The major strength of the paper is the use of data on the complete universe of hospital discharges at all public Portuguese hospitals, for a disease associated with a very high morbidity and mortality burden. The large and comprehensive database contributes to the validity of our findings about the precise contribution of new technologies to health expenditures.

### Implications

The literature has long demonstrated the relevance of new technologies in improving populations’ survival and quality of life, and as driver of health expenditures. This prompted the need to carefully evaluate the value for money of new technologies, in a context of multiple competing interests in the healthcare sector, through the development of HTA agencies in most high-income countries. This paper contributes first to justify the development of HTA for new drugs, which indeed strongly contribute to health expenditures growth, i.e., more than a quarter of it in our specific case. Second, the paper shows that administrative data can be reliably used as a source of real-world evidence in HTA, provided data are large and representative, and adequate statistical techniques are used.

To our knowledge, this research indeed provides a novel perspective in understanding the drivers (contributors) of healthcare expenditure growth by being the first attempt to apply the verified Blinder–Oaxaca decomposition approach to quantify the contributions of expenditure drivers. In this way, this research provides the best evidence available on the effect of technology advancements on public healthcare expenditure in Portugal using the case of IHD.

## Supplementary Information

Below is the link to the electronic supplementary material.

Supplementary file1 (DOCX 44 KB)
