# Supplementary material for: Contribution of high-technology procedures to public healthcare expenditures: the case of ischemic heart disease in Portugal, 2002–2015
Source: Int J Health Econ Manag. 2024 Mar 29;24(3):419–37. doi: 10.1007/s10754-024-09372-5 (PMC11445372; doi:10.1007/s10754-024-09372-5)
Supplement: Supplementary file 1 — Supplementary file1 (DOCX 44 KB) [file 10754_2024_9372_MOESM1_ESM.docx]

## Supplementary Materials

### Recent studies on technology and healthcare expenditure

**Table S 1 Recent studies on the association between technology and healthcare expenditure. Methods for analysis including type of the model used, model outcome, and technological factor and other covariates considered are presented for each study.**

| **Publication** | **Country / Region** | **Study Period** | **Disease Indication** | **Method / Model** | **Outcome*** | **Technological Factor*** | **Other Covariates Included*** |
| --- | --- | --- | --- | --- | --- | --- | --- |
| Cantarero 2010 [13] | Spain | 1993 - 1999 | All diseases | Panel data models | Per capita healthcare expenditure | Two alternative proxies: time trend and a set of time fixed effects | Per capita income, density of acute care beds, density of general practitioners, demographic structure |
| Abrantes-Metz 2012 [11] | US | 1965 - 2005 | All diseases | Regression analysis | Per capita healthcare expenditure | Residual | Real per capita administrative costs, real per capita personal income, real health care price, CMS share, population share over 65 |
| Chandra 2012 [5] | OECD countries | 1980 - 2008 | All diseases | Models for patient demand and supplier behaviour | -- | Three types of medical technology: highly cost-effective innovations with little chance of overuse, treatments highly effective for some but not for all, and treatments with uncertain clinical value | -- |
| Bilgel and Tran 2013 [14] | Canada | 1975 - 2002 | All diseases | Fixed effect models | Per capita government healthcare expenditure | Linear trend | Per capita GDP, population structure, life expectancy, relative price of health care |
| Hartwig and Sturm 2014 [18] | 33 OECD countries | 1970 - 2010 | All diseases | Extreme bound analysis | Growth of real per capita healthcare expenditure | 15 technological and capacity factors identified from literature review, e.g., density of acute beds and total expenditures on R&D | All other determinants of HCE growth in that have been suggested in the literature, e.g., social-demographic factors, institutional factors, prices, and variables needed to build explanatory variables |
| Wu 2014 [12] | 16 OECD countries | 1975 - 2009 | All diseases | Panel smooth transition regression models | Per capita healthcare expenditure | Time trend | GDP, time, lagged ratio of public expenditures on health |
| Murthy 2016 [16] | US | 1960 - 2012 | All diseases | Autoregressive distributed lag cointegration approach | Per capita real US health spending | Health R&D expenditure | Population percent above 65, income, age, health care technology exert positive effects |
| Murthy 2017 [9] | US | 1960 - 2012 | All diseases | Cointegration analysis | Per capita healthcare expenditure | Four proxies: Real health R&D expenditure, total R&D expenditure in the economy, per capita real health R&D expenditure, per capita real total R&D expenditure | Per capita real income, life expectancy at birth |
| Nghiem 2017 [8] | OECD countries | 1975 - 2004 | All diseases | Fixed effect models | Per capita healthcare expenditure | Time trend | Real GDP per capita, proportion of population over 65, share of public expenditure in total health spending, country-specific fixed effects |
| You 2017 [15] | Australia | 1971 - 2011 | All diseases | Cointegration analysis | Per capita total expenditure on health | 5 alternative measures: Per capita R&D, per capita hospital research expenditures, unweighted and weighted technology indexes constructed from medical devices, and infant mortality rate | Disposable income per capita, age > 65 population ratio |
| Liu 2020 [17] | Taiwan | 1997 - 2006 | Type 2 diabetes | Decomposition method | Growth in health expenditure | Two components: treatment substitution and treatment expansion | The effect of population ageing (prevalence rate and disease severity) |
| Sorenson 2013 [56] | -- | -- | Cardiovascular diseases | Literature review | -- | -- | -- |
| Achdut 2019 [53] | Israel | -- | All diseases | Literature review | -- | -- | -- |
| Amiri 2021 [54] | -- | Until 2017 | All diseases | Systematic review | -- | -- | -- |

Abbreviations: US = United States, OECD = Organisation for Economic Co-operation and Development, R&D = research and development, GDP = gross domestic product

*The natural logarithm form for costs were commonly used for modelling, CMS = Centres for Medicare & Medicaid Services

### Change in time trend of healthcare expenditure

The following tables present detailed results of the regression of per capita healthcare expenditure on year dummies for all IHD patients, and patients with AMI, UA, and SA.

**Table S 2 Yearly change in per capita healthcare expenditure for all IHD patients. Among all patients under study, for each year starting from 2003 the association between that year’s per capita healthcare expenditure and that for the previous year was tested to identify any significant change in per capita expenditure between the two years.**

| Year | Coefficient | SE | t | Prob > t | 95% CI Lower | 95% CI Upper | F (Coeff [Year] = Coeff [Year - 1]) | Prob > F |
| --- | --- | --- | --- | --- | --- | --- | --- | --- |
| 2003 | 151.92 | 39.88 | 3.81 | <.001 | 73.75 | 230.08 | - | - |
| 2004 | 476.34 | 39.89 | 11.94 | <.001 | 398.16 | 554.52 | 66.01 | <.0001 |
| 2005 | 595.68 | 40.27 | 14.79 | <.001 | 516.74 | 674.61 | 8.76 | 0.0031 |
| 2006 | 645.70 | 40.01 | 16.14 | <.001 | 567.28 | 724.12 | 1.53 | 0.2162 |
| 2007 | 360.15 | 38.30 | 9.40 | <.001 | 285.09 | 435.22 | 55.05 | <.0001 |
| 2008 | 904.59 | 38.09 | 23.75 | <.001 | 829.94 | 979.24 | 222.74 | <.0002 |
| 2009 | 1084.87 | 38.74 | 28.00 | <.001 | 1008.94 | 1160.81 | 23.82 | <.0003 |
| 2010 | 1252.68 | 38.95 | 32.16 | <.001 | 1176.34 | 1329.01 | 19.68 | <.0004 |
| 2011 | 1428.02 | 39.18 | 36.45 | <.001 | 1351.23 | 1504.81 | 20.99 | <.0005 |
| 2012 | 1669.67 | 39.28 | 42.51 | <.001 | 1592.68 | 1746.65 | 39.16 | <.0006 |
| 2013 | 1697.71 | 39.06 | 43.46 | <.001 | 1621.15 | 1774.26 | 0.53 | 0.4663 |
| 2014 | 1023.28 | 39.05 | 26.20 | <.001 | 946.74 | 1099.82 | 310.74 | <.0001 |
| 2015 | 945.55 | 39.03 | 24.22 | <.001 | 869.04 | 1022.05 | 4.13 | 0.042 |
| _cons | 2561.17 | 28.17 | 90.93 | <.001 | 2505.96 | 2616.38 | - | - |

Abbreviations: SE = standard error, CI = confidence interval

**Table S 3 Yearly change in per capita healthcare expenditure for AMI patients. Among patients diagnosed with AMI, for each year starting from 2003 the association between that year’s per capita healthcare expenditure and that for the previous year was tested to identify any significant change in per capita expenditure between the two years.**

| Year | Coefficient | SE | t | Prob > t | 95% CI Lower | 95% CI Upper | F (Coeff [Year] = Coeff [Year - 1]) | Prob > F |
| --- | --- | --- | --- | --- | --- | --- | --- | --- |
| 2003 | 197.27 | 64.49 | 3.06 | 0.002 | 70.87 | 323.67 | - | - |
| 2004 | 570.62 | 64.58 | 8.84 | <.001 | 444.04 | 697.21 | 34.81 | <.0001 |
| 2005 | 707.81 | 65.21 | 10.85 | <.001 | 580.00 | 835.61 | 4.59 | 0.0321 |
| 2006 | 822.89 | 65.03 | 12.65 | <.001 | 695.42 | 950.35 | 3.19 | 0.0742 |
| 2007 | 958.70 | 64.40 | 14.89 | <.001 | 832.47 | 1084.93 | 4.55 | 0.0329 |
| 2008 | 1932.28 | 63.97 | 30.21 | <.001 | 1806.90 | 2057.66 | 242.14 | <.0001 |
| 2009 | 2151.37 | 64.43 | 33.39 | <.001 | 2025.09 | 2277.65 | 12.25 | 0.0005 |
| 2010 | 2118.53 | 64.39 | 32.90 | <.001 | 1992.32 | 2244.75 | 0.27 | 0.6024 |
| 2011 | 2364.11 | 64.48 | 36.67 | <.001 | 2237.74 | 2490.48 | 15.16 | 0.0001 |
| 2012 | 2581.96 | 64.16 | 40.24 | <.001 | 2456.20 | 2707.71 | 12.02 | 0.0005 |
| 2013 | 2605.16 | 63.95 | 40.73 | <.001 | 2479.82 | 2730.51 | 0.14 | 0.7095 |
| 2014 | 1734.45 | 63.82 | 27.18 | <.001 | 1609.35 | 1859.54 | 197.53 | <.0001 |
| 2015 | 1638.76 | 63.51 | 25.80 | <.001 | 1514.29 | 1763.24 | 2.42 | 0.1197 |
| _cons | 2846.77 | 46.50 | 61.21 | <.001 | 2755.62 | 2937.92 | - | - |

Abbreviations: SE. = standard error, CI = confidence interval

**Table S 4 Yearly change in per capita healthcare expenditure for UA patients. Among patients diagnosed with UA, for each year starting from 2003 the association between that year’s per capita healthcare expenditure and that for the previous year was tested to identify any significant change in per capita expenditure between the two years.**

| Year | Coefficient | SE | t | Prob > t | 95% CI Lower | 95% CI Upper | F (Coeff [Year] = Coeff [Year - 1]) | Prob > F |
| --- | --- | --- | --- | --- | --- | --- | --- | --- |
| 2003 | 160.60 | 53.72 | 2.99 | 0.003 | 55.31 | 265.88 | - | - |
| 2004 | 335.79 | 55.33 | 6.07 | <.001 | 227.33 | 444.25 | 9.25 | 0.0024 |
| 2005 | 359.86 | 58.45 | 6.16 | <.001 | 245.30 | 474.42 | 0.15 | 0.698 |
| 2006 | 391.37 | 60.62 | 6.46 | <.001 | 272.55 | 510.19 | 0.22 | 0.637 |
| 2007 | 538.74 | 59.27 | 9.09 | <.001 | 422.56 | 654.92 | 4.76 | 0.0291 |
| 2008 | 840.97 | 59.53 | 14.13 | <.001 | 724.29 | 957.65 | 20.63 | <.0001 |
| 2009 | 928.34 | 64.04 | 14.50 | <.001 | 802.82 | 1053.87 | 1.52 | 0.2173 |
| 2010 | 759.49 | 67.70 | 11.22 | <.001 | 626.79 | 892.20 | 4.71 | 0.03 |
| 2011 | 922.61 | 68.12 | 13.54 | <.001 | 789.09 | 1056.12 | 4.03 | 0.0446 |
| 2012 | 948.98 | 65.15 | 14.57 | <.001 | 821.29 | 1076.67 | 0.11 | 0.7388 |
| 2013 | 891.67 | 61.83 | 14.42 | <.001 | 770.48 | 1012.85 | 0.6 | 0.437 |
| 2014 | 799.31 | 61.44 | 13.01 | <.001 | 678.88 | 919.74 | 1.72 | 0.1901 |
| 2015 | 849.90 | 60.43 | 14.06 | <.001 | 731.45 | 968.35 | 0.53 | 0.4651 |
| _cons | 1668.09 | 36.26 | 46.00 | <.001 | 1597.02 | 1739.17 | - | - |

Abbreviations: SE = standard error, CI = confidence interval

**Table S 5 Yearly change in per capita healthcare expenditure for SA patients. Among patients diagnosed with SA, for each year starting from 2003 the association between that year’s per capita healthcare expenditure and that for the previous year was tested to identify any significant change in per capita expenditure between the two years.**

| Year | Coefficient | SE | t | Prob > t | 95% CI Lower | 95% CI Upper | F (Coeff [Year] = Coeff [Year - 1]) | Prob > F |
| --- | --- | --- | --- | --- | --- | --- | --- | --- |
| 2003 | -17.82 | 155.88 | -0.11 | 0.909 | -323.35 | 287.72 | - | - |
| 2004 | 49.07 | 155.74 | 0.32 | 0.753 | -256.19 | 354.33 | 0.18 | 0.6696 |
| 2005 | 129.64 | 157.97 | 0.82 | 0.412 | -179.98 | 439.26 | 0.26 | 0.612 |
| 2006 | 82.30 | 149.22 | 0.55 | 0.581 | -210.17 | 374.77 | 0.1 | 0.7562 |
| 2007 | -121.37 | 136.98 | -0.89 | 0.376 | -389.85 | 147.12 | 2.43 | 0.1188 |
| 2008 | 372.92 | 135.26 | 2.76 | 0.006 | 107.81 | 638.02 | 18.68 | <.0001 |
| 2009 | 160.86 | 142.01 | 1.13 | 0.257 | -117.49 | 439.20 | 3.11 | 0.078 |
| 2010 | 124.98 | 150.19 | 0.83 | 0.405 | -169.40 | 419.35 | 0.07 | 0.7933 |
| 2011 | 232.56 | 150.98 | 1.54 | 0.123 | -63.36 | 528.49 | 0.54 | 0.4618 |
| 2012 | 269.33 | 152.85 | 1.76 | 0.078 | -30.27 | 568.92 | 0.06 | 0.805 |
| 2013 | 365.17 | 157.39 | 2.32 | 0.02 | 56.69 | 673.66 | 0.38 | 0.5374 |
| 2014 | 354.23 | 155.39 | 2.28 | 0.023 | 49.66 | 658.80 | 0.00 | 0.9448 |
| 2015 | 344.22 | 151.92 | 2.27 | 0.023 | 46.46 | 641.98 | 0.00 | 0.9476 |
| _cons | 1271.43 | 109.50 | 11.61 | <.001 | 1056.80 | 1486.05 | - | - |

Abbreviations: SE = standard error, CI = confidence interval
